# Mechanisms of Human Motor Learning Do Not Function Independently

**DOI:** 10.3389/fnhum.2021.785992

**Published:** 2022-01-04

**Authors:** Amanda S. Therrien, Aaron L. Wong

**Affiliations:** Moss Rehabilitation Research Institute, Elkins Park, PA, United States

**Keywords:** cerebellar degeneration, adaptation, reinforcement learning, explicit and implicit motor learning, sensory prediction error

## Abstract

Human motor learning is governed by a suite of interacting mechanisms each one of which modifies behavior in distinct ways and rely on different neural circuits. In recent years, much attention has been given to one type of motor learning, called motor adaptation. Here, the field has generally focused on the interactions of three mechanisms: sensory prediction error SPE-driven, explicit (strategy-based), and reinforcement learning. Studies of these mechanisms have largely treated them as modular, aiming to model how the outputs of each are combined in the production of overt behavior. However, when examined closely the results of some studies also suggest the existence of additional interactions between the sub-components of each learning mechanism. In this perspective, we propose that these sub-component interactions represent a critical means through which different motor learning mechanisms are combined to produce movement; understanding such interactions is critical to advancing our knowledge of how humans learn new behaviors. We review current literature studying interactions between SPE-driven, explicit, and reinforcement mechanisms of motor learning. We then present evidence of sub-component interactions between SPE-driven and reinforcement learning as well as between SPE-driven and explicit learning from studies of people with cerebellar degeneration. Finally, we discuss the implications of interactions between learning mechanism sub-components for future research in human motor learning.

## Introduction

The field of motor neuroscience has greatly advanced our understanding of how humans learn to produce and control new movements. There are many contexts in which motor learning occurs, such as when learning to perform movements *de novo* or learning the appropriate sequence of movements necessary to execute a skilled action. Here, we focus on studies of a third motor learning context, often termed motor adaptation, in which one must learn to modify an existing movement pattern to account for persistent changes to the body, task, or environmental dynamics (Krakauer et al., 2019). All types of motor learning likely rely on multiple interacting mechanisms that, in turn, rely on different neural circuits. However, the mechanisms underlying motor adaptation have received particular attention in recent years, with most literature studying the interactions between three mechanisms: learning driven by sensory prediction errors (SPEs, or the difference between the sensory outcome of a movement and a prediction of that outcome), explicit (or strategy-based) learning, and reinforcement (or reward-based) learning. Studies of interactions between these mechanisms have largely treated them as modular, focusing on how each mechanism’s outputs are combined to produce overt learning behavior. To isolate one or more learning mechanisms, studies have modified the attentional cues and/or sensory feedback provided during behavioral learning tasks. Intriguingly, these manipulations have produced evidence of additional interactions between the sub-components of the different learning mechanisms. Here, we propose that understanding these sub-component interactions is needed to advance our knowledge of how learning mechanisms combine to produce overt behavior. We first summarize the current literature studying interactions between SPE-driven, explicit, and reinforcement mechanisms of motor learning. We then present evidence of sub-component interactions between SPE-driven and reinforcement learning, as well as between SPE-driven and explicit learning, from studies of people with cerebellar degeneration. We conclude with a discussion of considerations for future research.

### Motor Adaptation Results From the Interaction of Multiple Mechanisms

**Figure 1:**
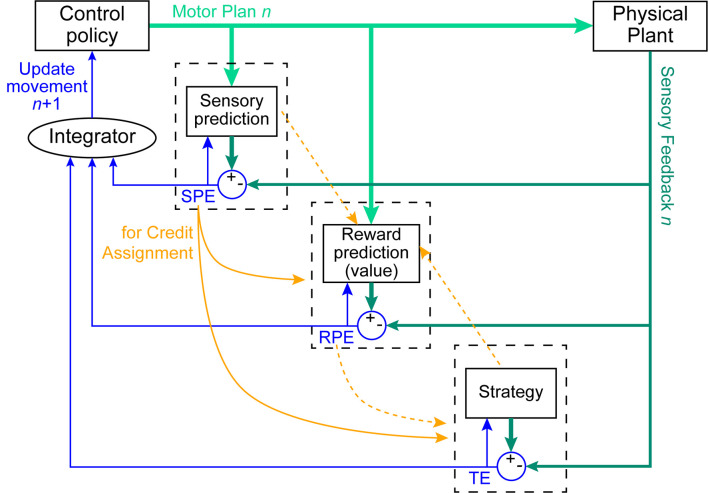
Control policy updates arising from the interactions of three learning mechanisms. On trial *n*, a control policy is issued to perform the current movement (light green thick arrows). This plan is executed by the body (physical plant), and sensory feedback is detected (dark green arrows). The SPE-driven learning system predicts the expected sensory consequences of the movement, which is compared against sensory feedback of the actual executed movement to compute a sensory prediction error (SPE). The reinforcement learning system predicts the expected reward associated with that movement and this is compared against the actual reward outcome to compute a reward prediction error (RPE). The explicit learning system compares the expected outcome of the strategy against the observed movement outcome to compute a task error (TE). In all cases, the computed error signals (thin blue arrows) update both the respective prediction mechanism as well as the control policy for the next (*n* + 1) movement. Most studies treat this control-policy update as the combination of the contributions of the individual learning systems (here labeled as the Integrator). We suggest that these systems also interact in other ways. For example, SPE signals are a means by which the reinforcement-learning and explicit-learning systems could solve the credit-assignment problem in determining whether the policy or the execution of that policy led to the observed result (solid orange arrows). Additional speculated interactions may exist (dashed orange arrows), although more behavioral evidence is needed to support the existence of such connections in humans.

While several mechanisms have been proposed to contribute to motor learning, three have largely been assumed to account for the vast majority of observed behavioral changes in simple motor adaptation tasks (Krakauer et al., 2019). These three mechanisms are SPE-driven learning, explicit learning, and reinforcement learning (Figure 1). Each of these mechanisms is thought to respond to a different kind of feedback signal, and consequently, drive changes in behavior in different (and occasionally opposing) ways and at different rates (Mazzoni and Krakauer, 2006; van der Kooij et al., 2018; Albert et al., 2020; Morehead and Orban de Xivry, 2021). In general, the study of these mechanisms has treated them as modular, typically assuming that observed behavior can be described as the summation of the outputs of the individual mechanisms. Thus, when the contribution of a single mechanism cannot be easily isolated experimentally, it is often estimated by subtracting out the influence of a second, more easily measured mechanism (Taylor et al., 2014; McDougle et al., 2015; Maresch et al., 2021).

One commonly used task to study motor adaptation has participants generate a movement such as a reach or a saccade toward a target. Participants are then presented with a predictable perturbation that alters the outcome of that movement, which necessitates learning to alter the movement pattern to account for the imposed perturbation. For example, in a task requiring the adaptation of reaching movements to a visuomotor rotation, individuals observe a cursor move at a fixed non-zero angle relative to their actual hand motion, which is hidden from view. Over many trials, participants learn to adjust their motor plans to reach in a direction opposite the perturbation to reduce the error. Trial-to-trial learning in this adaptation task has been shown to be supported by all three mechanisms.

SPE-driven learning was the first mechanism recognized to contribute to behavioral changes in adaptation tasks. SPEs convey the difference between the sensory outcome of a movement and a prediction of that outcome based on a copy of the outgoing motor command (Kawato, 1999; Tseng et al., 2007; Shadmehr et al., 2010; Morehead et al., 2017). The SPE signal is thought to be computed by the cerebellum (Medina, 2011; Schlerf et al., 2012); hence people with cerebellar damage are known to exhibit poor performance in adaptation tasks (Criscimagna-Hemminger et al., 2010; Izawa et al., 2012; Therrien et al., 2016; Wong et al., 2019). SPEs do not necessarily reflect task failure, but rather the fact that a movement did not result in the predicted sensory outcome according to the planned motor command. Thus, if an inappropriate motor command was executed accurately (e.g., reaching toward the target when the task is to reach in the opposite direction from the target), it would result in a task error but not an SPE. More recently, such task errors (specifically, the observed difference between the movement outcome and the intended movement target or goal) have also been suggested to drive learning under this mechanism (Miyamoto et al., 2014; Leow et al., 2018; Albert et al., 2020). Regardless, SPE-driven learning requires sensory information about the direction and magnitude (i.e., vector information) of movement errors. In motor adaptation tasks, vector error information is typically provided by contrasting the desired target location with a visual representation of the index fingertip position during reaching movements (e.g., a cursor on a screen). The signature of SPE-driven learning (and the most reliable measure of its impact on behavior) is the existence of after-effects—behavioral changes reflecting a new mapping of motor commands to predicted sensory outcomes that persist even after the perturbation has been removed. SPE-driven learning is described as occurring without conscious awareness, possibly due to a concomitant recalibration of perception (Ostry and Gribble, 2016; Rossi et al., 2021), and can be expressed even at low reaction times (approximately 130 ms, Haith et al., 2015; Leow et al., 2017; Hadjiosif and Krakauer, 2021); hence, it is often referred to as implicit learning. By most accounts, SPE-driven learning is thought to be the primary driving force behind motor adaptation (Izawa and Shadmehr, 2011; Therrien et al., 2016; Cashaback et al., 2017; Wong et al., 2019).

In addition to SPE-driven learning, prior work has emphasized a large contribution of an explicit learning mechanism. In the context of adaptation tasks, explicit learning is often described as the acquisition of an aiming strategy or learning to deliberately move somewhere other than the target location. For example, if a cursor is rotated 45° clockwise relative to the hand, people can accurately move their hand to a target if they adopt a strategy of aiming their reach 45° counterclockwise from the target. Broadly speaking, explicit learning arises as a result of a task error (i.e., awareness that the task objective was not achieved), although exactly how task errors are quantified and how they lead to changes in behavior are not well understood. Nevertheless, studies probing the relationship between SPE-driven and explicit learning often assume that these mechanisms have an additive impact on behavior (Mazzoni and Krakauer, 2006; Benson et al., 2011; McDougle et al., 2015; Long et al., 2016; Miyamoto et al., 2020). Researchers often subtract explicit aiming reports from net learning to measure SPE-driven learning (e.g., Taylor et al., 2014). Alternatively, researchers might measure the SPE-driven learning process using a process dissociation procedure and subtract it from net learning to estimate the contribution of an explicit process (Werner et al., 2015). Many studies have used these methods to examine adaptation across the age span and have suggested that impaired performance in older individuals is largely due to a reduced contribution of the explicit learning mechanism, while the SPE-driven learning system remains intact (McNay and Willingham, 1998; Bock, 2005; Heuer and Hegele, 2008; Hegele and Heuer, 2013; Vandevoorde and Orban de Xivry, 2019).

Finally, there is reinforcement learning. Despite being one of the earliest learning mechanisms to have been studied in the context of behavior modification (Thorndike, 1905), studies have only recently begun to carefully examine its contribution to adaptation tasks. Reinforcement learning occurs in response to scalar feedback about performance outcomes. In the extreme case, scalar feedback may be a binary signal (e.g., an auditory tone indicating success or failure), but reinforcement learning can also occur in response to a success gradient (e.g., hot/cold). Studies of motor adaptation have attempted to leverage reinforcement learning by providing binary or gradient feedback in place of a visual cursor representing the position of the hand during reaching movements. In this way, an individual does not have access to the direction or magnitude of movement errors; rather, the individual must explore possible task solutions to discern those that yield success. Reinforcement learning induces a change in behavior by increasing the likelihood of generating movements associated with rewarding outcomes. It is thought to depend on reward-prediction errors (RPEs), computed in midbrain dopaminergic circuits, which convey the difference between predicted and actual rewards (Schultz, 2006; Lee et al., 2012). Although learning in response to rewards could occur as part of a deliberate decision-making strategy, here we classify such situations as examples of explicit learning since they are primarily driven by task errors (where the “task” in this case is to choose the most rewarding option). Instead, we view reinforcement learning as an implicit process, in line with the notion that behavioral conditioning can occur without needing to explicitly learn the relationship between stimulus, response, and outcome (Skinner, 1937). Indeed, in motor learning tasks, exploration of the response space (characteristic of a reinforcement learning process) can be driven by unconscious motor variability (Wu et al., 2014), and reinforcement learning has been shown to couple with other implicit processes such as use-dependent learning (Mawase et al., 2017). However, more work is needed to carefully dissociate the explicit and implicit effects of learning in response to reinforcement.

Reinforcement learning can occur either as a stand-alone process that is independent of the other learning mechanisms, or by interacting with either the SPE-driven or explicit process. In the former case, reinforcement learning drives motor learning without recalibrating perception (Izawa and Shadmehr, 2011). It may operate by inducing both exploration of the reward landscape as well as the repetition of more successful movements (Nikooyan and Ahmed, 2015; Cashaback et al., 2017; Uehara et al., 2019). Thus, reinforcement learning may complement other learning mechanisms by contributing in an additive manner to the net observed behavior (Kim et al., 2019), although if only scalar feedback is provided this could alternatively reduce the amount of learning arising from another mechanism like SPE-driven learning (Izawa and Shadmehr, 2011; van der Kooij et al., 2018). On the other hand, reinforcement learning may have a more intimate interaction with SPE-driven or explicit learning. It could do so by increasing the likelihood of selecting more successful behaviors that have been identified through these other learning mechanisms (Shmuelof et al., 2012; Nikooyan and Ahmed, 2015). For example, reinforcement learning may help individuals to identify and preferentially select more successful explicit strategies (Bond and Taylor, 2015; Codol et al., 2018; Holland et al., 2018; Rmus et al., 2021) because the explicitly-identified action also becomes associated with greater reward. Regardless of its exact mechanism of action, reinforcement learning is typically treated as acting in conjunction with other learning mechanisms to modify behavior (Haith and Krakauer, 2013).

### Evidence of Interactions Between Sub-components of Learning Mechanisms

Although the interactions between SPE-driven, explicit, and reinforcement learning mechanisms have largely been modeled as a summation or integration of each mechanism’s outputs, imperfect additivity has been noted (e.g., Maresch et al., 2021). Deviations from model predictions have sometimes been attributed to additional learning processes not measured or, alternatively, to the inability of measurement methods to fully capture a given mechanism’s output. However, some work suggests the additional possibility that sub-components of each mechanism may also interact. That is, the computations underlying one learning mechanism may serve a critical role in the functioning of another. Understanding the nature of sub-component interactions is crucial, as their presence significantly complicates attempts to experimentally parse the contribution of different learning mechanisms in behavioral tasks. To date, the clearest evidence of sub-component interactions comes from studies of people with cerebellar degeneration. With the cerebellum’s role in SPE-driven learning well established, one hypothesis has been that cerebellar damage selectively disrupts this learning mechanism. Yet studies attempting to distinguish SPE-driven, explicit, and reinforcement learning in people with cerebellar degeneration have not shown the hypothesized dissociation (McDougle et al., 2016; Therrien et al., 2016; Wong et al., 2019).

Therrien et al. (2016) attempted to distinguish supervised and reinforcement learning in people with cerebellar degeneration by modifying error feedback in an adaptation task. In one condition, SPE-driven learning was leveraged by providing full vector feedback of movement errors in the form of a visual cursor representing the index fingertip position throughout reaching movements. In a second condition, reinforcement learning was leveraged by providing only binary feedback of reach success or failure. People with cerebellar degeneration showed distinct behaviors in the two learning conditions: no retention of learning (i.e., no after-effect) when provided with vector error feedback, but significant retention when provided with binary feedback. If examined only at the output level of each mechanism, these results are consistent with cerebellar degeneration impairing supervised learning and leaving reinforcement learning intact. However, people with cerebellar degeneration learned more slowly with binary feedback compared to age-matched control participants, suggesting that cerebellar degeneration may reduce the efficiency of reinforcement learning. Importantly, this latter result pointed to a previously unknown interaction between cerebellar computations and reinforcement learning.

**Figure 2:**
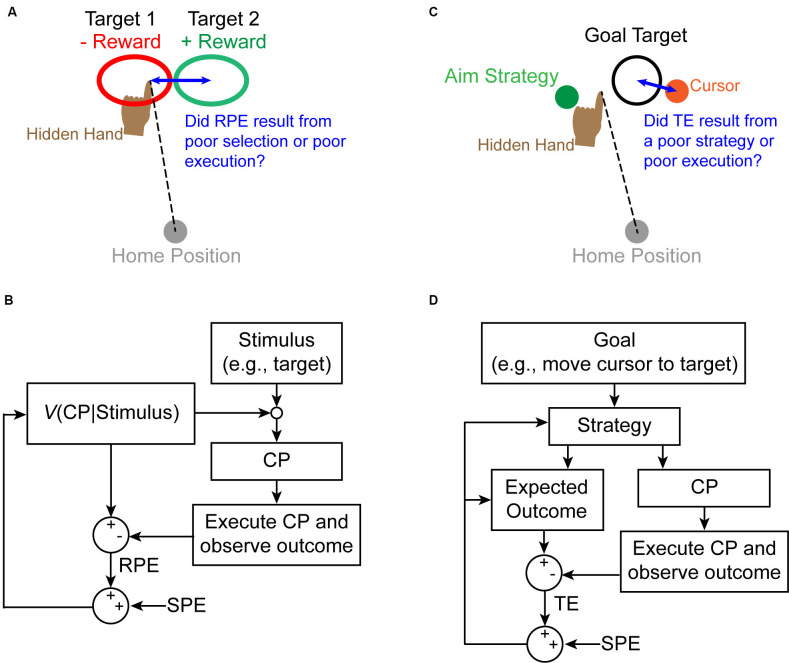
Proposed interactions between the SPE signal and other learning mechanisms to solve the credit-assignment problem. **(A)** On a given trial, individuals receive positive or negative reward feedback about reach outcome. If this feedback is unexpectedly negative (i.e., a negative RPE signal), for example, individuals must determine whether they erroneously selected the wrong control policy or simply executed the correct policy poorly. **(B)** An example state diagram corresponding to the situation in panel **(A)** describes how an update signal is generated based on an RPE (indicating an error has occurred). An SPE is used to determine if the RPE should be attributed to a poor policy choice or a poor execution of that policy. **(C)** During explicit learning, an individual adopts a strategy (e.g., aim location) to attain a goal (hit the target with the cursor). If a task error arises, individuals must determine if they erroneously selected the wrong explicit strategy or if they poorly executed the correct strategy. **(D)** Although it remains unclear exactly how explicit learning occurs, we propose that updates to the strategy choice occur as a result of a task error (TE), which is modulated by an SPE informing about the accuracy of executing the chosen strategy.

How could cerebellar computations contribute to reinforcement learning? Cerebellar SPEs may be used to solve reinforcement learning’s credit-assignment problem (Taylor and Ivry, 2014; McDougle et al., 2016; Therrien et al., 2016, 2018). In reinforcement learning, the valence of RPE signals is used to update the future probability of selecting a particular motor response to a given stimulus (Dayan and Niv, 2008; Haith and Krakauer, 2013). However, motor response execution is rife with uncertainty due to a combination of noise inherent in the sensorimotor system and variable properties of the environment (Franklin and Wolpert, 2011). Sensorimotor uncertainty makes determining the true cause of reward signals (i.e., credit-assignment) ambiguous. Cerebellar SPEs convey whether a movement was executed as intended, and thus constitute a particularly useful solution to the credit-assignment problem (Figures 2A,B).

Reinforcement learning behavior is known to account for higher-order statistical properties of sensorimotor uncertainty, such as the distribution standard deviation (Trommershäuser et al., 2008; Wu et al., 2009, 2014; Landy et al., 2012). However, behavioral variability reflects variance in both motor planning (i.e., response selection) and motor execution (van Beers et al., 2004; van Beers, 2009). Therrien et al. (2016); Therrien et al. (2018) modeled reinforcement learning with behavioral variability parsed into exploration, representing planning variability, and motor noise, representing execution variability. Their conjecture was that, after positive reinforcement, response selection is updated in a manner that accounts for exploration, but not motor noise. In their studies, people with cerebellar degeneration displayed reinforcement learning behavior consistent with excessive variability being allotted to motor noise—a pattern indicative of impaired estimation of action execution. People with cerebellar degeneration also showed reduced exploration after negative reinforcement (Therrien et al., 2018), suggesting that cerebellar degeneration impacts the integration of both positive and negative reinforcement signals. The cumulative result is a reduced updating of action selection in response to reinforcement signaling that slows learning in this population.

McDougle et al. (2016) examined the role of SPE-like sensorimotor error signals in solving a credit-assignment problem in reinforcement-based decision making. Participants were required to select between two visual targets, each associated with a different magnitude of reward, by reaching to hit one or the other. On some trials they were given false feedback about the accuracy of their reach, which generated RPEs—the actual reward received differed from the expected outcome. In contrast to neurologically healthy participants, people with cerebellar degeneration were unable to determine if RPEs should be attributed to themselves or the experimental manipulation (i.e., solve the credit-assignment problem) in this task, suggesting that reach-related sensorimotor error signals play an important role in reinforcement learning.

Reinforcement learning is not the only situation in which a credit-assignment problem must be resolved. Although it is less clear exactly how explicit learning operates, sensorimotor uncertainty likely contributes to a credit-assignment problem similar to that identified above. For example, one must determine if an error arose because of a poor choice of strategy, or because of poor execution of the chosen strategy. Here again, the involvement of an SPE signal would be beneficial to formulate and modify explicit strategies by informing how well the intended strategy was executed (Figures 2C,D).

Evidence supporting the involvement of an SPE-like signal in explicit learning arises from a series of studies investigating the ability of people with cerebellar degeneration to develop *de novo* strategies for learning. As noted above, cerebellar degeneration disrupts the signal supporting SPE-driven learning, which impairs performance during a visuomotor rotation paradigm. Previous work had demonstrated that in such tasks, people with cerebellar degeneration could follow a provided strategy to aim in a direction other than the target (i.e., opposite the visuomotor rotation), allowing them to overcome the perturbation and successfully hit the target (Taylor et al., 2010). Such an observation led to a puzzling question—if their ability to employ strategies was so successful, why did not people with cerebellar degeneration use strategies all the time to compensate for their movement deficits instead of continuing to rely on an impaired SPE-driven learning system? Butcher et al. (2017) showed that, on their own, people with cerebellar degeneration had great difficulty invoking explicit learning to identify a successful aiming strategy that would minimize task errors. That is, some people with cerebellar degeneration continued to aim directly for the target despite the presence of the visuomotor rotation perturbation. However, Wong et al. (2019) revealed that this was only part of the answer. Under certain circumstances, people with cerebellar degeneration could successfully develop *de novo* strategies using explicit learning. Wong and colleagues demonstrated that when people with cerebellar degeneration were able to view their actual hand moving simultaneously with the cursor, they could resolve the credit assignment problem by recognizing that task errors were not a result of a mis-executed motor command but instead caused by a manipulation of the cursor. That is, people with cerebellar degeneration could use visual feedback to appropriately attribute performance errors to task errors rather than execution errors. Consequently, people with cerebellar degeneration were able to invoke explicit learning to modify their movement goals (i.e., change their aiming direction) akin to that of age-matched neurotypical controls. This work thus suggests a role for SPE signals in supporting explicit learning. While more work is needed to parse the specific role that such SPE signals may play, together these studies provide compelling evidence of interactions between cerebellar computations and both explicit and reinforcement learning mechanisms.

## Conclusion

We have reviewed current literature on the interactions between SPE-driven, explicit, and reinforcement learning mechanisms in motor adaptation. It is generally agreed that overt learning behavior results from the combined outputs of each mechanism, but interactions between these mechanisms likely occur at multiple levels. For example, studies of people with cerebellar degeneration provide evidence of a role for SPE signals in the functioning of both reinforcement and explicit learning. These studies suggest that an SPE signal may be needed by reinforcement and explicit learning systems to know whether RPEs or task errors, respectively, arose from poorly executed movements or poor selection of an action or strategy. By helping to resolve this credit-assignment problem, SPEs can optimize learning by informing reinforcement and explicit learning systems whether an action or strategy truly needs to change.

It is notable that some of the neuroanatomy needed to support these proposed interactions has been shown. With regard to a role for cerebellar SPE signals in reinforcement learning, the cerebellum communicates directly with the dorsal striatum *via* a short-latency disynaptic connection that modulates corticostriatal plasticity (Hoshi et al., 2005; Chen et al., 2014). The posterior lobules of the cerebellum are also reciprocally connected with prefrontal cognitive regions of the cerebral cortex, which are hypothesized to support the explicit learning process (Ramnani, 2006; Strick et al., 2009). The nature of the information sent through these pathways is unclear, but there is recent evidence to suggest homologous function across cerebellar projections (Pisano et al., 2021). However, the cerebellum contributes to a diverse set of behaviors, both motor and non-motor (Diedrichsen et al., 2019; King et al., 2019; Sereno et al., 2020). Further work is needed to understand whether different regions of the cerebellum may be preferentially involved in the interactions proposed here or whether variability in the pattern of cerebellar damage across individuals and studies can explain some contrasting results. Sharing of the SPE signal represents one of the multiple possible interactions among SPE-driven, reinforcement, and explicit learning mechanisms below the level of their output stages (see Figure 1), and future research is needed to elucidate others. Importantly, the presence of such multi-level interactions means that learning mechanisms cannot be easily isolated.

When it comes to motor adaptation, studies of people with cerebellar degeneration suggest that SPE-driven learning may be the primary system responsible for resolving performance errors. Only when the influence of SPE-driven is minimized, such as by eliminating the need or ability to compute a meaningful SPE signal (e.g., by removing cursor feedback altogether or providing visual feedback of the hand), can reinforcement learning or explicit learning become the predominant driver of changes in behavior (Therrien et al., 2016, 2021; Cashaback et al., 2017; Wong et al., 2019). This has important implications for future studies aiming to manipulate or leverage individual learning mechanisms.

Finally, the work reviewed here begs the question of whether further insight into the interactions between SPE-driven, explicit, and reinforcement learning mechanisms can be gained from studies of motor adaptation in other patient populations. Parkinson’s disease (PD) is often studied as a model of basal ganglia dysfunction, a structure known to play an integral role in reinforcement learning (Schultz, 2006; Lee et al., 2012). A sizable body of literature has studied motor adaptation in people with PD but has noted inconsistent findings. While some studies show similar adaptation behavior between people with PD and age-matched control participants (e.g., Stern et al., 1988; Marinelli et al., 2009; Leow et al., 2012, 2013), other studies have noted adaptation impairments in people with PD (Contreras-Vidal and Buch, 2003; Venkatakrishnan et al., 2011; Mongeon et al., 2013). Discrepant results may stem from differences in the size of the imposed perturbation (Venkatakrishnan et al., 2011; Mongeon et al., 2013) or medication status of participants across studies (Semrau et al., 2014). To date, no study has attempted to parse the contributions of SPE-driven, explicit, and reinforcement learning to motor adaptation in this population (but see Cressman et al., 2021), but it would be highly interesting for future studies to do so. Overall, this literature, along with the other studies reviewed here, underscores the complexity of interactions occurring between motor learning mechanisms and argues for the importance of not treating such learning mechanisms as predominantly modular.

## Author Contributions

Both AT and AW contributed equally to development of the idea, writing the manuscript, and generation of figures. All authors contributed to the article and approved the submitted version.

## Conflict of Interest

The authors declare that the research was conducted in the absence of any commercial or financial relationships that could be construed as a potential conflict of interest.

## Publisher’s Note

All claims expressed in this article are solely those of the authors and do not necessarily represent those of their affiliated organizations, or those of the publisher, the editors and the reviewers. Any product that may be evaluated in this article, or claim that may be made by its manufacturer, is not guaranteed or endorsed by the publisher.
